# Allergic Asthma-Induced Cognitive Impairment is Alleviated by Dexamethasone

**DOI:** 10.3389/fphar.2021.680815

**Published:** 2021-06-23

**Authors:** Mengli Ren, Min Feng, Zhimin Long, Jing Ma, Xuehua Peng, Guiqiong He

**Affiliations:** ^1^ Institute of Neuroscience, Basic Medical College, Chongqing Medical University, Chongqing, China; ^2^ Department of Anatomy, Basic Medical College, Chongqing Medical University, Chongqing, China; ^3^ Wuhan Children’s Hospital (Wuhan Maternal and Child Healthcare Hospital), Tongji Medical College, Huazhong University of Science and Technology, Wuhan, China

**Keywords:** allergic asthma, cognitive function, dexamethasone, oxygen deficit, house dust mites

## Abstract

Allergic asthma is a typical chronic inflammatory disease of respiratory tract. Clinical data shows that patients with allergic asthma have different degrees of cognitive dysfunction. The molecular mechanism underlying the pathogenesis of asthma-induced cognitive disorder is not yet well defined. Dexamethasone (DEX), one of the first-line drugs being widely used in the treatment of asthma, has not been reported to have an effect on cognitive dysfunction in mice model. To investigate the effect of asthma on cognitive impairment as well as the effect of DEX on asthma-caused morphological and behavioral changes, C57BL/6J mice received treatment with house dust mites (HDM) for 60 days to become allergic asthma model mice, and a group of HDM-treated asthma model mice were treated with DEX. HDM-treated asthma model mice exhibited increased airway hyperresponsiveness (AHR) and inflammatory infiltration in lung tissue. An elevated level of IL-4, IL-5, and TNF-α was detected in bronchoalveolar lavage fluid (BALF) by Luminex liquid suspension chip. Asthma model mice also presented memory deficits accompanied with morphological changes at the synaptic levels in the cortex and hippocampus. Meanwhile, vascular edema and increased expression of HIF-1α and HIF-2α were found in the brain of asthma model mice. Interestingly, DEX treatment could reverse the inflammatory changes in asthma model mice airway, rescue the cognitive impairment and improve the synaptic plasticity. Besides, DEX significantly decreased the expression of HIF-1α and HIF-2α in mice brain and lung. These processes may be used to decipher the complex interplay and pathological changes between asthma and cognition. This study provides laboratory evidence for the prevention and treatment of cognitive malfunction induced by asthma.

## Introduction

Allergic asthma is a highly heterogeneous airway disease induced by exposure to environmental triggers and is characterized by airway inflammation, AHR, elevated immunoglobulin (Ig) E level, and airway remodeling accompanied by clinical symptoms such as wheezing, shortness of breath, chest tightness, cough, and restricted airflow (Tyler and Bunyavanich, 2019). About 300 million people around the world now suffer from asthma, with allergic asthma expected to increase to 400 million by 2025 (Price, 2008). According to a national survey conducted by the China Asthma Alliance in 2013, the total prevalence of asthma in China reached 1.24 percent. Another survey, published in 2017, put the number of asthmatics in China at nearly 30 million, or 2 percent of the population. In some cases, allergic asthma disease can seriously damage the patient’s physical and mental health, affecting the patient’s quality of life and bringing heavy burden to the patient’s family and society. Clinical studies suggest that a large part (less than fifty percent) of asthmatic patients have different degree of cognitive dysfunction, particularly those with more severe or longer duration of the disease (Irani et al., 2017; Rhyou and Nam, 2021). Cognitive dysfunction is a condition in which abnormalities in the brain’s higher intellectual processing lead to severe learning and memory problems (Harvey, 2019).

The brain is the organ with the largest consumption of oxygen, accounting for 20% of the total. Compared with other tissues and organs, the brain is more sensitive to oxygen, so hypoxia becomes one of the important risk factors leading to cognitive dysfunction. It is reported that when oxygen concentration is as low as 15–16%, cognitive function will be impaired to a certain extent (Qaid et al., 2017). In patients with respiratory diseases associated with hypoxemia, 77% of the population suffer from cognitive dysfunction. Hypoxia inducible factor (HIF) is the main transcription factor and regulator of genomic hypoxia response (Ema et al., 1997; Yu et al., 2017). The key pathological feature of asthma is AHR, which is prevalently caused by airway inflammation (Brannan and Lougheed, 2012). Bronchial stenosis caused by allergic asthma can lead to varying degrees of hyoxemia (Vargas Becerra, 2009). During asthma attacks, hypoxemia may reduce the amount of oxygen to be used by vital organs.

DEX is an oral corticosteroid that can be used in the treatment of patients affected by severe uncontrolled asthma when biological therapies are not indicated. Studies have shown that moderate DEX treatment reduces allergen-induced asthma models, reduces the expression of inflammatory factors in BALF and reduces infiltration of peribronchial inflammatory cells (Guan et al., 2020). To date, there is a lack of laboratory literature about the relationship between asthma and cognition and about the effect of DEX on asthma-related cognitive dysfunction in mice model.

**FIGURE 1:**
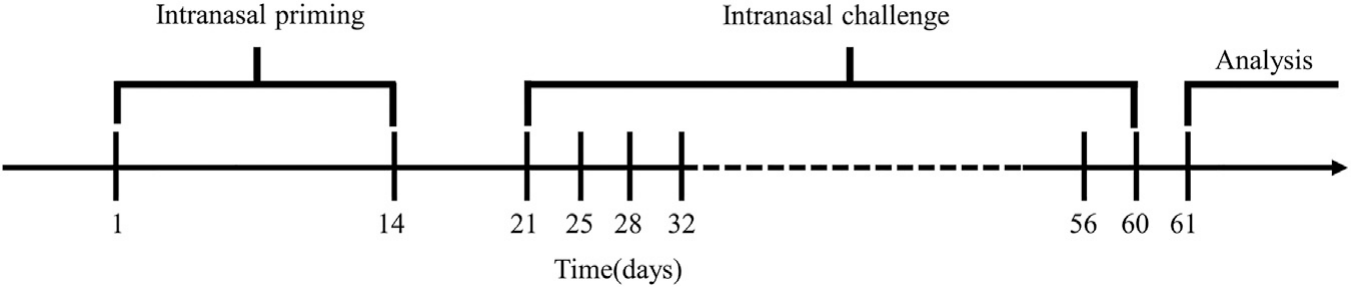
Model building cycle. Mice were sensitized from day 1 to day 14, stimulated twice a week for six consecutive weeks from day 21 to day 60, and lung function and behavioral tests were performed on day 61.

In this study, we successfully established a model of mice with asthma caused by house dust mites (HDM) for 60 days (Figure 1). Our *in vivo* data demonstrated that HDM-treated asthma model mice showed significantly cognitive decline and neuropathological changes in mice brain which may have been caused by increased airway inflammatory response and following cerebral hypoxia. DEX could greatly reverse the worsening of cognition and synaptic function *via* anti-inflammation of respiratory tract and alleviate the hypoxia status of the brain. This study may provide a basis for prevention and treatment of asthma-related neuropathogenesis and cognitive impairment.

## Materials and Methods

### Animals

Animal care and experimental procedures in this study were in accordance with the guidelines for the Care and Use of Laboratory Animals from Ethics Committee of Chongqing Medical University. 6–8 weeks old C57BL/6J mice were housed and acclimated to standard laboratory conditions (12 h light/dark cycle; lights on at 7:00 AM and off at 7:00 PM) with free access to fodder and water.

### Allergic Asthma Model Construction/Drug Treatment

After screening the learning and memory abilities through the Morris water maze test (see supplementary Supplementary Figure S1), 40 mice were divided into four groups: Control group, NaCl group, HDM group and DEX group (*n* = 10 in each group). Control group mice received no nasal drops. NaCl group mice were treated with vehicle solution 0.9% NaCl. To induce asthma, these mice were sensitized to 20 μg of HDM (Greer, Los Angeles, CA, United States) in 30 μl normal saline (NS) *via* nasal inhalation (Zou et al., 2019). The duration of nasal drops was divided into two stages: the sensitization stage and the excitation stage. Nasal drops were performed on day 1 and day 14 of the sensitization stage. The excitation stage lasts for 6 weeks, and nasal drops were performed twice a week (Locke et al., 2007). DEX group mice were administered through orogastric gavage following the previous studies (Kumar et al., 2003; Babayigit et al., 2009) with 1 mg/kg DEX (sigma, Germany) at 1 h before HDM inhalation.

### Lung Function Analysis

Lung function in all the mice was measured within 24 h after the last excitation stage. AHR was detected to evaluate airway function using erosolized methacholine. Different doses (0, 3.125, 6.25, 12.5, 25, and 50 mg/ml) of methacholine (sigma, Germany) induced airway contraction in mice by inhalation challenge. All the mice were put into an experimental chamber to record the enhanced pause (Penh) for detected AHR values using a whole-body plethysmograph (EMKA, France).

### Barnes Maze

At the end of the model cycle, Barnes maze task was used to test the spatial learning and memory abilities in accordance with the method used by Pompl et al. (1999) but with minor modifications (Souza et al., 2012). The Barnes maze apparatus consists of a 122 cm diameter disk and 18 circular holes (5 cm diameter) rising 100 cm above the ground. The escape chest (13*29*14 cm) was placed under a hole. The device was surrounded by curtains and decorated with different bright signs. Animals were trained in the Barnes maze on the first day for one trial. The training was to put the mice in an escape box and then leave for a minute. Then, the escape box was placed in the center of the Barnes maze. The escape box was removed and the training began. The mice searched for the escape box under the hole freely in the maze. The longest delay time of finding the escape box was 300 s, and the time of finding the escape box and the number of wrong holes were measured. After the first day of training, the test began, and the data for two trials were recorded over the next 5 days. On the seventh day, the escape box was removed and each mouse was placed in the center of the maze. The number of times the observed mice entered the escape box hole and other holes was recorded.

### Preparation of Tissue

At the end of treatment, the mice were euthanized by CO_2_ inhalation. Mice were perfused transcardially with 0.01 M phosphate buffered saline (MPBS) (pH 7.4). The brain was divided into the left cerebral hemisphere and the right cerebral hemisphere. The left cerebral hemisphere was immediately homogenized for Western blotting (WB), and the right cerebral hemisphere was post-fixed in freshly prepared 4% formalin buffer for immunofluorescent staining. The rest cerebral hemisphere was bluntly dissected to the hippocampus and cortex for TEM. All procedures were performed on ice.

For HE staining, left lung tissues of all the mice were fixed in 4% formalin buffer and embedded in paraffin. The paraffin blocks were then serially sectioned into 4 μm-thick slices and subjected to HE staining.

For immunofluorescent staining, excised brains were immersed in 4% formalin buffer overnight, 20% sucrose for 24 h and 30% sucrose for another 48 h. Then, the brains were embedded in optimum cutting temperature compound in a freezing microtome, and 10 mm-thick sections were cut with a freezing microtome (Leica, Germany).

For TEM, mice from each group were transcardially perfused with 0.01 MPBS, followed by 2.5% glutaraldehyde-4% paraformaldehyde in 0.01 MPBS. The brain was quickly stripped in an ice bath and 1 mm^3^ tissue sections were cut from the hippocampus in the CA1 area and the cortex.

### Hematoxylin Eosin Staining

The paraffin section of lung tissue was dewaxed and hydrated. The staining procedure was then performed according to the HE staining kit (SolarBio, Beijing, China).

### Immunofluorescence Staining

The sections were incubated with blocking reagent (Beyotime Biotechnology, Shanghai, China) for 1 h. Brain sections and lung sections from each mice group were incubated with the following primary antibodies: anti-HIF-1α (1:200, Bioss, Beijing, China), anti-HIF-2α (1:200, Bioss, Beijing, China), at 4°C overnight. The sections were then incubated with secondary antibodies (1:5,000, Proteintech, Wuhan, China) at 37°C for 1 h after a thorough wash. The nuclei were stained with DAPI (Solarbio, Beijing, China) and placed at room temperature for 5 min. Anti-fluorescence quenching agent (Solarbio, Beijing, China) was added to seal the tablet and was observed under fluorescence microscope.

### TEM

The brain tissues were fixed in 2.5% electron microscopy-specialized glutaraldehyde for 2 h, washed several times with 0.01 MPBS, post-fixed in 1% osmium tetroxide for 2 h, and dehydrated with gradient solutions. The brain tissues were embedded with Epon812 epoxy resin. The brain tissue blocks were then cut into 1 μm semi-thin sections, placed on slides, stained with azure-methylene blue, and examined with a light microscope. The brain tissues were sectioned on a Leica EM UC6 ultramicrotome at 60–80 nm and collected on pioloform-coated Cu2*1 oval slot grids (Electron Microscopy Sciences, Hatfield, PA, United States).

### Luminex Liquid Suspension Chip

The mouse chest was opened and the right main bronchus was ligated. Ophthalmic scissors were used to cut an oblique opening on the trachea, intubation was performed, and then lavage with 0.3 ml of 0.1 MPBS was performed twice. The recovery rate of BALF was more than 80%. The assay was conducted according to manufacturer’s protocol using Bio-Plex Pro Mouse Cytokine Grp (#M60009RDPD) with Luminex 200 system (Austin, TX, United States) in Wayen Biotechnologies Shanghai, Inc.

### Western Blotting Analysis

The brain tissue protein extract and the lung tissue protein extract were prepared with rapamycin lysis buffer (RIPA; Beyotime Biotechnology, Shanghai, China) and phenylmethyl sulfonyl fluoride (PMSF) was added to it according to the instructions (Beyotime Biotechnology, Shanghai, China). The protein concentration was determined using a bicinchoninic acid (BCA) protein concentration kit (Beyotime Biotechnology, Shanghai, China). Equal amounts of proteins were resolved using a 10% SDS-PAGE gel kit (CWBIO, Beijing, China) and transferred onto polyvinylidene fluoride membranes (Millipore, United States). The membranes were incubated overnight at 4°C with the following primary antibodies: anti-HIF-1α (1:1,000, Abcam, United Kingdom), anti-HIF-2α (1:1,000, Abcam, United Kingdom) and anti-GAPDH (1:1,000, Affinity Biosciences, United States). After washing, the membranes were incubated for an hour at 37°C with horseradish peroxidase-conjugated secondary antibody (1:5,000, Proteintech, Wuhan, China). The immunoblots were visualized using enhanced chemiluminescence WB detection kits and then visualized using a molecular imager with Image Lab software (Bio-Rad, CA, United States). Protein bands were also quantified with Image Lab software (Bio-Rad, CA, United States).

### Statistical Methods

The data were expressed as mean ± SEM. GraphPad Prism software (Version 8.0; GraphPad, San Diego, Ca, United States) was used for statistical analysis. Statistical analysis for multiple comparisons was performed by two-way ANOVA, followed by Tukey’s multiple comparisons test. *P* value less than 0.05 was defined as statistically significant. All experimental analysis data were repeated at least three times and the results were consistent.

## Results

### The Allergic Asthma Mice Model was Successfully Constructed

**FIGURE 2:**
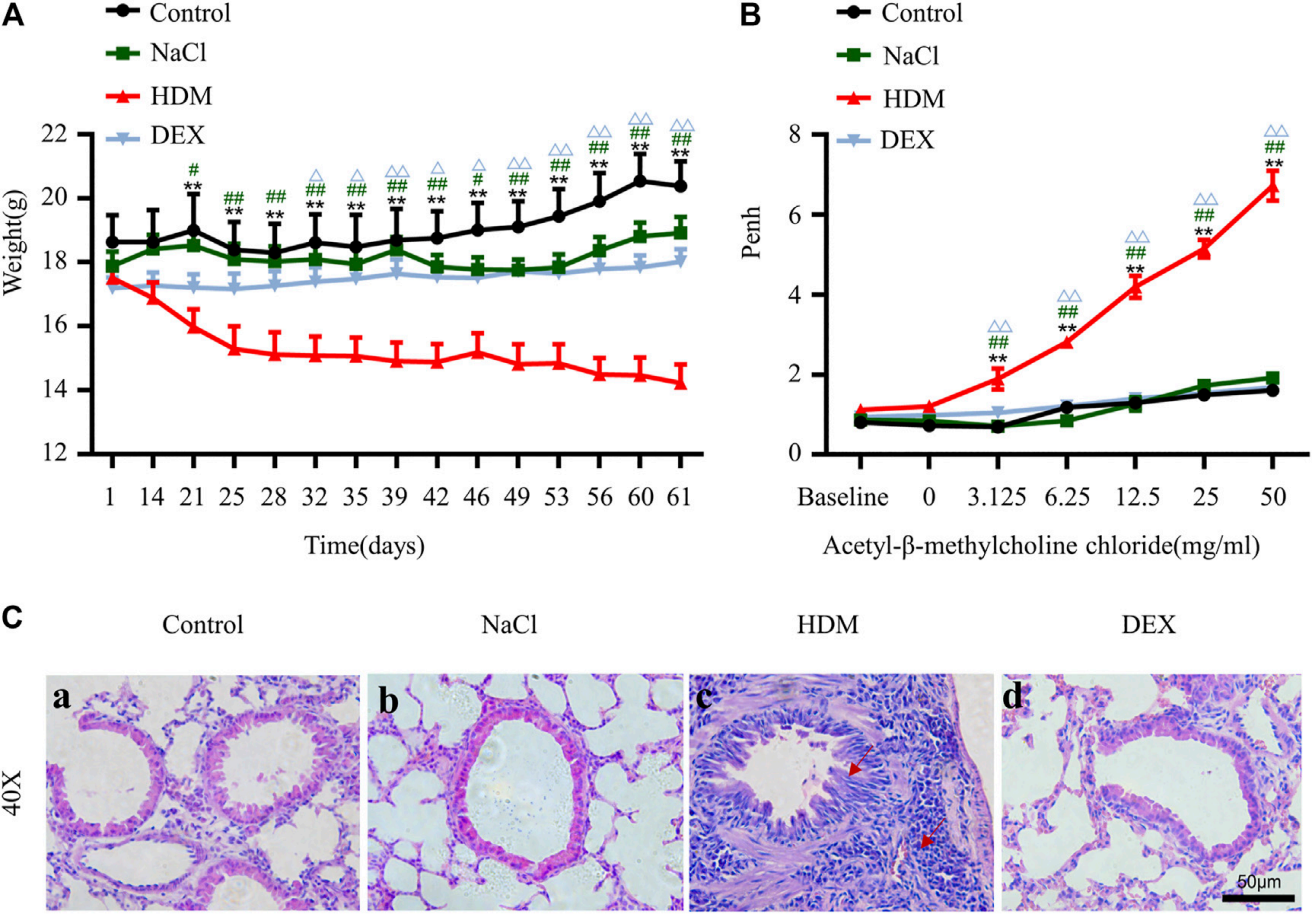
The allergic asthma mice model was established by house dust mite (HDM). **(A)** Changes in body weight during the model construction cycle. **(B)** Detection of airway hyperresponsiveness. **[C(a–d)]** HE staining of lung tissue. *n* = 8. *p < 0.05 and **p < 0.01 between HDM group and Control group. ^#^ *p* < 0.05 and ^##^ *p* < 0.01 between HDM group and NaCl group. ^△^ *p* < 0.05 and ^△△^ *p* < 0.01 between HDM group and DEX group.

**FIGURE 3:**
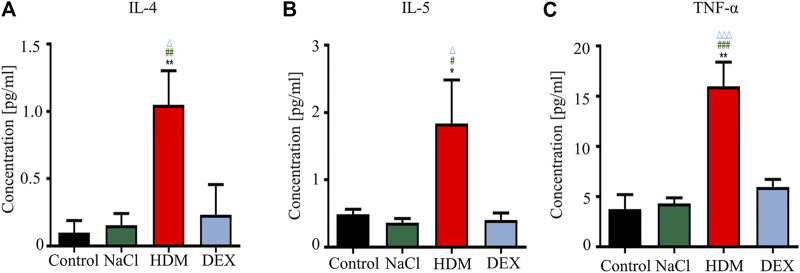
The concentrations of IL-4, IL-5, and TNF-α were increased in the BALF in the HDM group. **(A)** IL-4, **(B)** IL-5, **(C)** TNF-α. *p < 0.05 and **p < 0.01 between HDM group and Control group. *n* = 5. ^#^ *p* < 0.05, ^##^ *p* < 0.01 and ^###^ *p* < 0.001 between HDM group and NaCl group. ^△^ *p* < 0.05 and ^△△△^ *p* < 0.001 between HDM group and DEX group.

To verify the success of the allergic asthma mice model, we performed body weight (BW) monitoring, airway hyperresponsiveness measurement, and pulmonary histopathological assessment. The BW of mice was monitored during the model construction. The mice BW in the HDM group was significantly lower than that in the Control group (*p* < 0.01) and NaCl group (*p* < 0.05) on day 21 (Figure 2A). BWs of the DEX-treated (*p* < 0.05) were increased compared with HDM on day 32, but not identical to those of the control group. AHR is an important feature of respiratory function in asthma patients, and AHR was detected as Penh values (Gauvreau et al., 2015). The Penh values significantly increased in HDM group mice compared to the Control group (*p* < 0.01) and NaCl group (*p* < 0.01) at 3.125 mg/ml methacholine (Figure 2B). Tissue sections were stained with HE to observe eosinophil infiltration. There were significantly more inflammatory cells infiltrating the bronchus in the lungs of the asthmatic mice than in those of the normal mice (Figure 2C). DEX group mice could reduce eosinophil infiltration compared to HDM-sensitive mice. The Luminex liquid suspension chip was used to detect inflammatory cytokines in BALF. The inflammatory cytokines in HDM group were greatly increased compared with the Control group (IL-4, *p* < 0.01; IL-5, *p* < 0.05; TNF-α, *p* < 0.01) and the NaCl group (IL-4, *p* < 0.01; IL-5, *p* < 0.05; TNF-α, *p* < 0.001) (Figure 3). The inflammatory cytokines in DEX-treated mice were decreased, although they did not completely return to normal levels. These data indicated that HDM established in this study is an effective method for constructing the allergic asthma mice model.

### The Abilities of Spatial Learning and Memory was Decreased in the Allergic Asthma Mice Model

**FIGURE 4:**
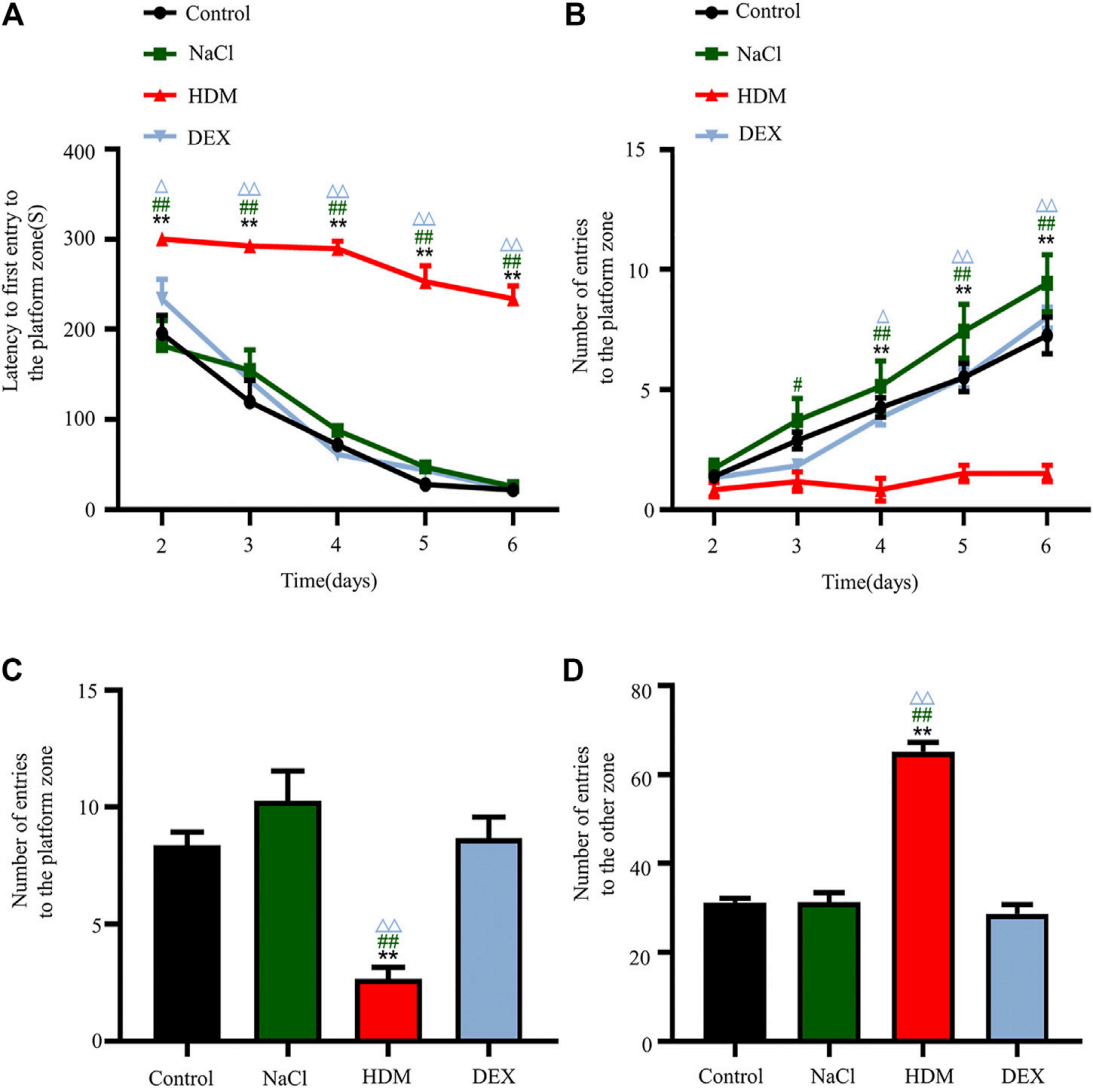
The cognitive ability of learning changed after modeling in the HDM group. **(A)** Latency to first entry to the platform zone in the Barnes maze experiment. **(B)** Number of entries to the platform zone in the Barnes maze training day. **(C)** Number of entries to the platform zone in the Barnes maze experiment. **(D)** Number of entries to the other zone in the Barnes maze experiment. *n* = 8. **p < 0.01 between HDM group and Control group. ^#^ *p* < 0.05 and ^##^ *p* < 0.01 between HDM group and NaCl group. ^△^ *p* < 0.05and^△△^ *p* < 0.01 between HDM group and DEX group.

To examine whether asthma affects spatial learning and memory, we performed the Barnes maze. After the adaptive training on the first day, the latency to first entry to the platform zone and the number of entries to the platform zone revealed the main effects of the next 5 days of training for the mice. The latency to first entry to the platform zone on day 2, 3, 4, 5, and 6 was longer in the HDM group than that in the Control group (*p* < 0.01), NaCl group (*p* < 0.01) and DEX group (*p* < 0.05) (Figure 4A). The number of entries to the platform zone in the HDM group was frequently smaller than that in the Control group (day 4, *p* < 0.01; day 5, *p* < 0.01; day 6, *p* < 0.01), NaCl group (day 3, *p* < 0.05; day 4, *p* < 0.01; day 5, *p* < 0.01; day 6, *p* < 0.01) and DEX group (day 4, *p* < 0.05; day 5, *p* < 0.01; day 6, *p* < 0.01) (Figure 4B). In the probe trial on the last day of the testing, HDM group mice had a decrease in the number of entries into the target hole (Figure 4C) and an increase in the number of entries into other holes (Figure 4D), when compared with Control group mice (*p* < 0.01), NaCl group mice (*p* < 0.01) and DEX group mice (*p* < 0.01). But there were some differences between the DEX group mice and the Control group mice. These data indicated that the HDM group mice significantly suffered from cognitive deficits.

### Destruction of Synaptic Structures, Cerebrovascular Edema and Collapse of Blood Vessels Were Observed in the Allergic Asthma Mice Model

**FIGURE 5:**
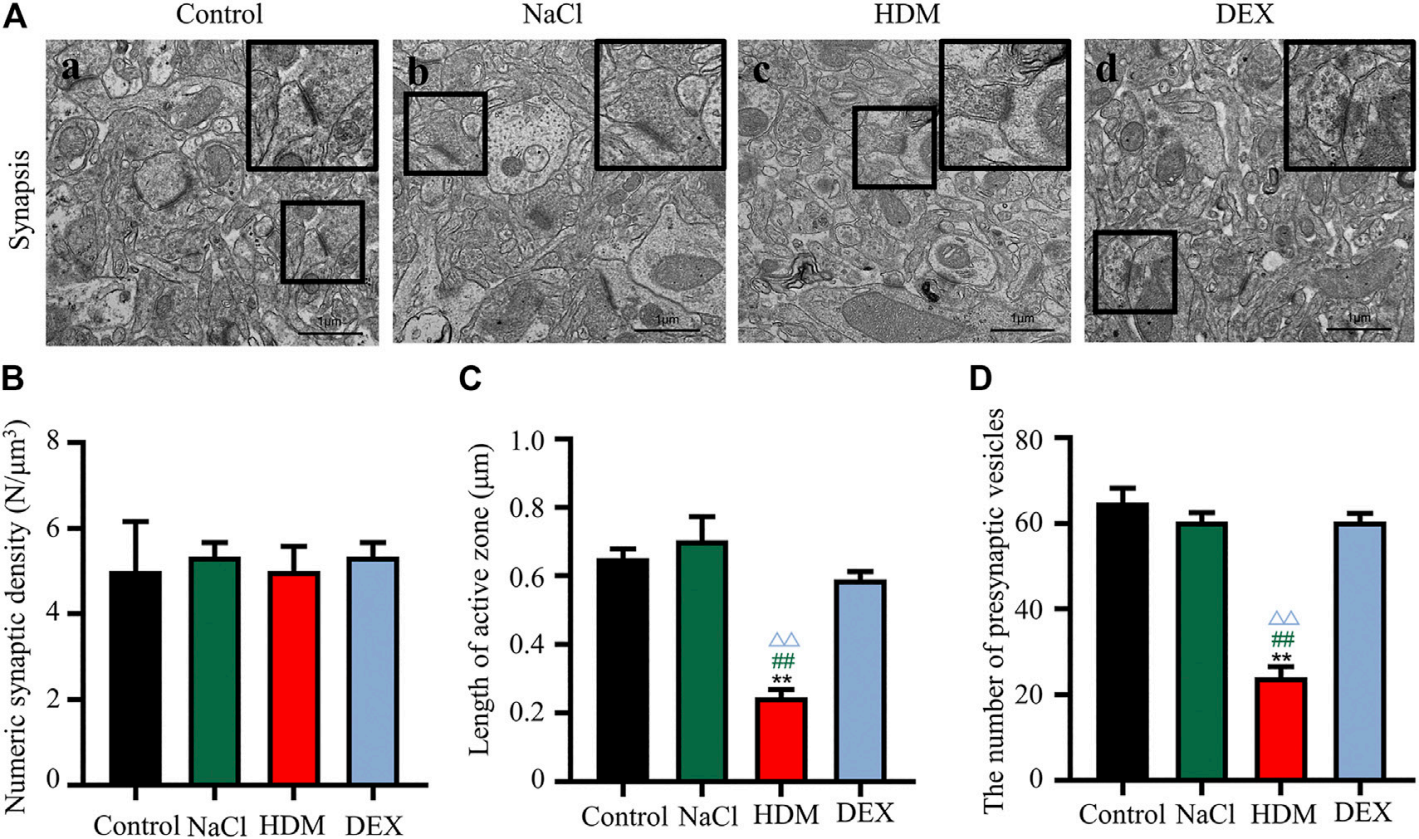
The synaptic activity length and the number of vesicles decreased in the hippocampus and cortex of mice in the HDM group. **[A(a–d)]** Brain tissue hippocampal synapses. **(B)** Numeric synaptic density. **(C)** Synapse length of active zone **(D)** The number of presynaptic vesicles. *n* = 5. **p < 0.01 between HDM group and Control group. ^##^ *p* < 0.01 between HDM group and NaCl group. ^△△^ *p* < 0.01 between HDM group and DEX group.

**FIGURE 6:**
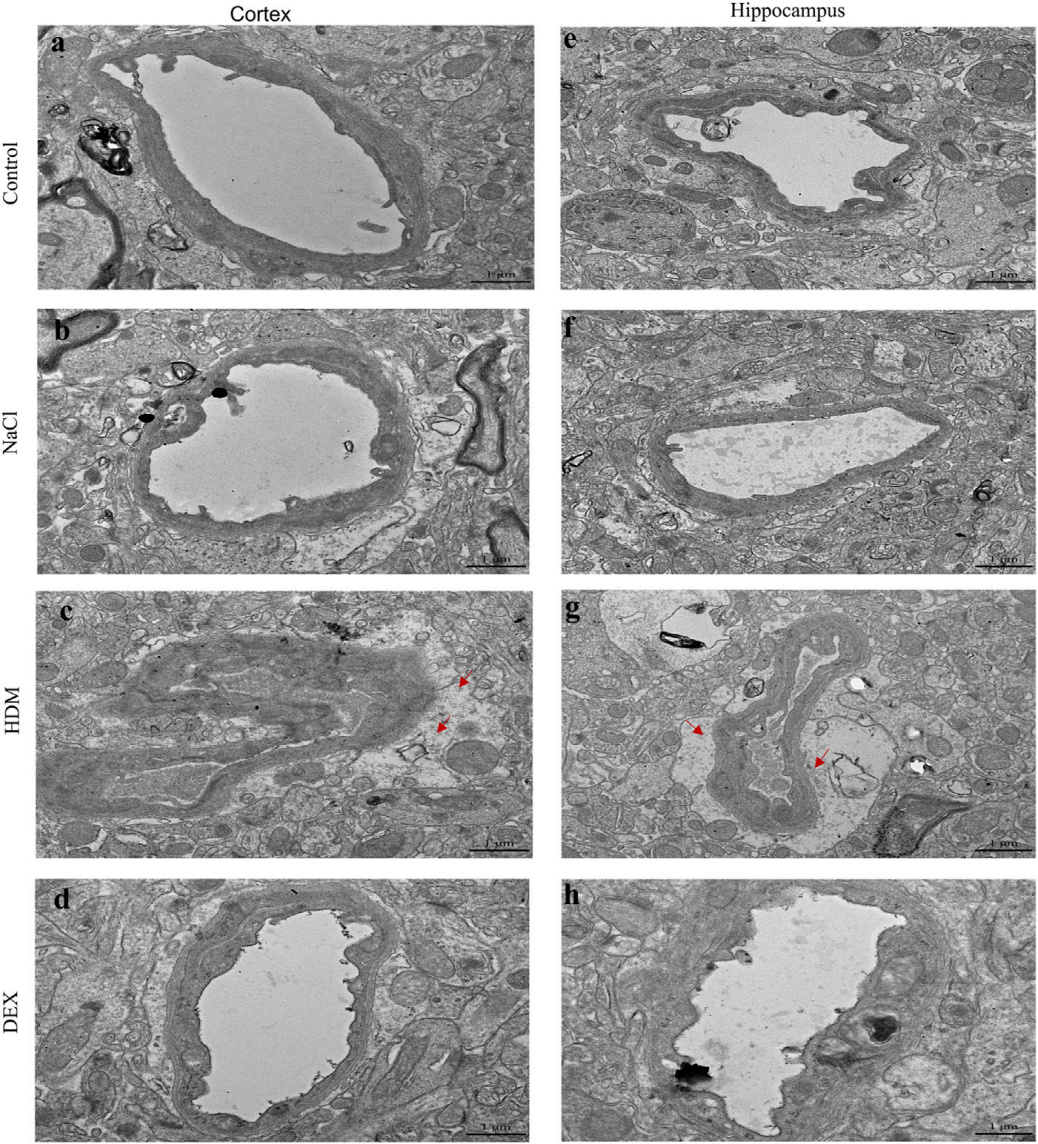
The blood vessels in the hippocampus and cortical area of the brain of mice in the HDM group were edema. **(a–d)** Blood vessels in the cortex of mice. **(e–h)** Blood vessels in the hippocampus of mice. The red arrow points to the area of vascular edema in the hippocampus and cortical area in the HDM group.

To confirm whether asthma causes learning and memory decline, we performed TEM to detect changes in the submicroscopic structure of the brain. Learning and memory are related to biochemical and morphological changes at the synaptic level. The number of synapses in the hippocampus of the HDM group mice was not statistically significant compared with that of the controls (Figure 5B). However, the length of active zone (Figures 5A,C) and the number of presynaptic vesicles (Figures 5A,D) of the HDM group mice were markedly decreased while compared to the Control group mice, the NaCl group mice and the DEX group. Furthermore, perivascular edema and vascular collapse appeared in the HDM group mice (Figure 6). These data indicated that the submicroscopic structure of brain of mice in HDM group was destroyed.

### The Expression of HIF-1α and HIF-2α was Increased in the Brain and Lung of the Allergic Asthma Mice Model

**FIGURE 7:**
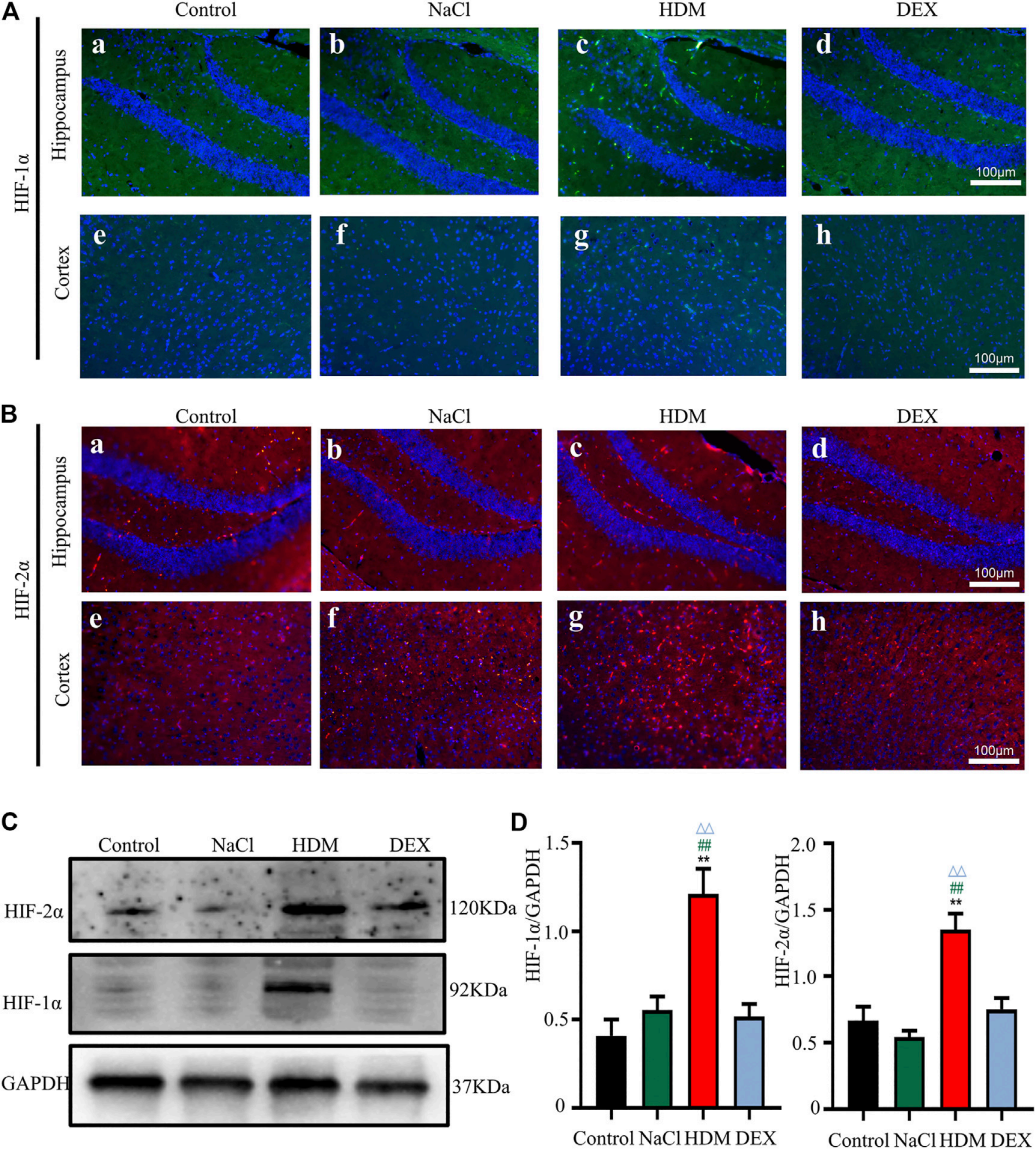
The expression of HIF-1α and HIF-2α in the brain was increased in the HDM group. **[A(a-h)]** Expression of HIF-1α in hippocampus and cortical regions of brain tissue. **[B(a-h)]** Expression of HIF-2α in hippocampus and cortical regions of brain tissue. **(C)** The protein expression of HIF-1α and HIF-2α in the brain tissues of mice was detected by Western blot. **(D)** The results of the western blot were quantified. *n* = 3. **p < 0.01 between HDM group and Control group. ^##^ *p* < 0.01 between HDM group and NaCl group. ^△△^ *p* < 0.01 between HDM group and DEX group.

**FIGURE 8:**
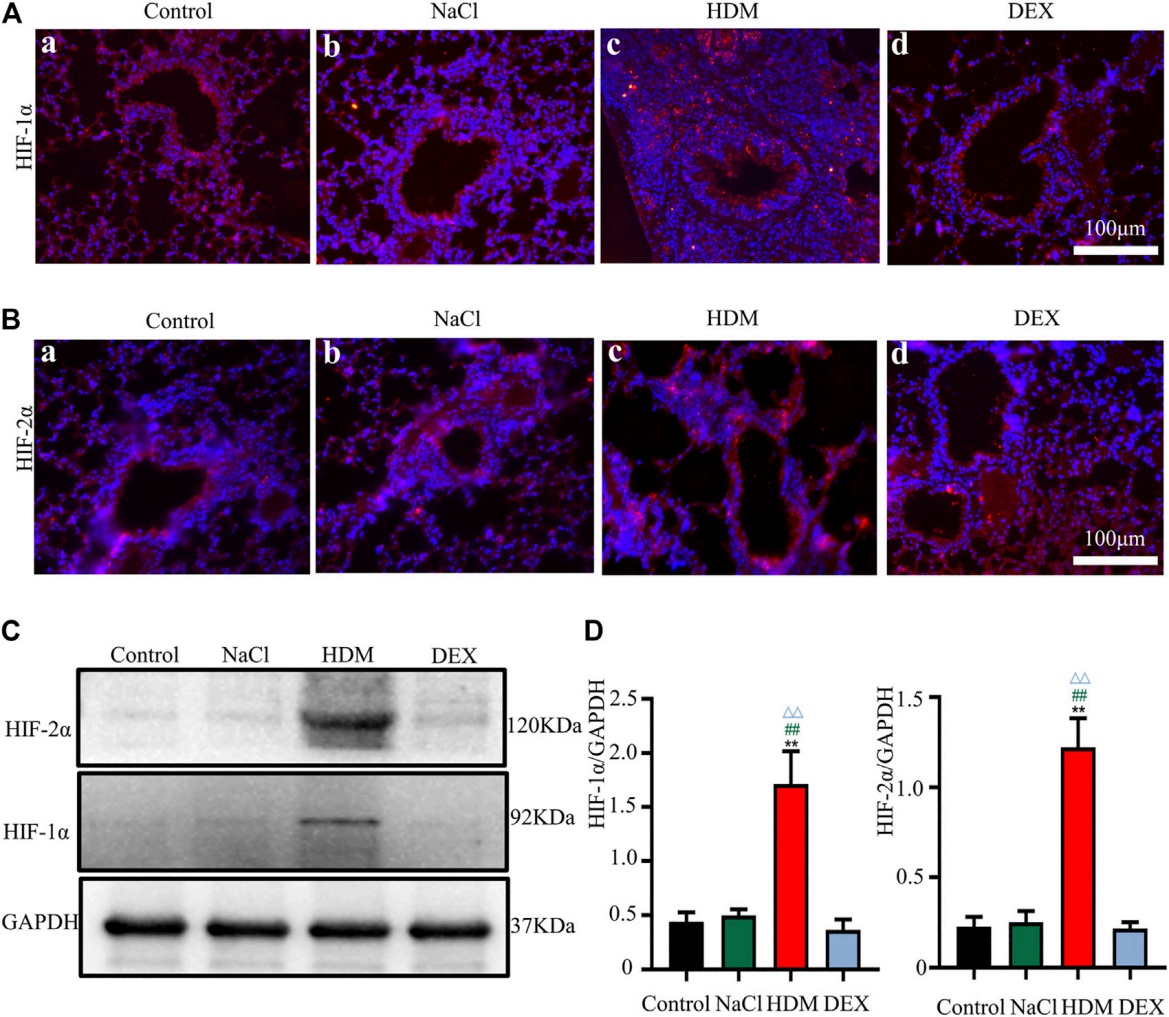
The expression of HIF-1α and HIF-2α in the lung was increased in the HDM group. **[A(a-d)]** Expression of HIF-1α in the lung tissue. **[B(a-d)]** Expression of HIF-2α in the lung tissue. **(C)** The protein expression of HIF-1α and HIF-2α in the lung tissues of mice was detected by Western blot. **(D)** The results of the western blot were quantified. *n* = 3. **p < 0.01 between HDM group and Control group. ^##^ *p* < 0.01 between HDM group and NaCl group. ^△△^ *p* < 0.01 between HDM group and DEX group.

Hypoxia may cause neurological deficits and structural damage to the brain tissue (Wilson et al., 2009). To further detect the oxygen levels in the brain and lung of asthmatic mice, we performed immunofluorescence staining and WB with the hypoxia-inducible factors marker HIF-1α and HIF-2α. Immunofluorescence analysis revealed that the HIF-1α and HIF-2α-positive cells in the brain and lung of HDM group mice were increased markedly (Figures 7A,B, 8A,B). Western blot analysis confirmed that the expression of HIF-1α and HIF-2α proteins in the brain of the HDM group mice was significantly elevated compared with the Control group (*p* < 0.01) and NaCl (*p* < 0.01) (Figures 7C,D). The expression of HIF-1α (*p* < 0.05) and HIF-2α (*p* < 0.05) of the DEX-treated mice was decreased compared with HDM, but not identical to the control group. Western blot analysis confirmed that the expression of HIF-1α and HIF-2α proteins in the lung of the HDM group mice was significantly increased compared with the Control group (*p* < 0.01), NaCl group (*p* < 0.01) and DEX group (*p* < 0.01) (Figures 8C,D). These data suggested that mice in the HDM group might suffer from cerebral hypoxia and pulmonary hypoxia.

## Discussion

Epidemiological investigations illustrated that individuals with asthma had a 78% increased risk for the presence of mild cognitive impairment (Caldera-Alvarado et al., 2013; Kroll et al., 2018). But laboratory literature about the relationship between asthma and cognition is lacking. The role of asthma in developing cognitive impairment need to be studied in animal models to prevent and treat the risk factors before they worsen. In this study, we constructed allergic asthma mice model by HDM. We found that the HDM group mice had inflammatory infiltration and AHR in the lung, the learning and memory abilities and hippocampal synaptic plasticity were decreased. Vascular edema and increased HIF-1α and HIF-2α expression were showed in the mice brain. However, DEX could alleviate the inflammatory changes and ventilation disorder of airway, decrease the cerebral hypoxia status, which in turn improve the cognitive impairment of the asthma model mice.

Since HDM represents the most common allergen which causes allergic asthma in the world (Aun et al., 2017), we had chosen HDM on C57BL/6 mice induced allergic asthma model for the validation studies. The allergic asthma mouse model was established by using HDM in accordance with the method of Zou et al. (2019) but with minor modifications (Locke et al., 2007). It was estimated that the antibodies against HDM allergens that 50–85% of all asthmatics carry were caused by HDM (Gehring et al., 2001; Bracken et al., 2015). C57BL/6 is susceptible to HDM and can be used to model allergic asthma induced by HDM (Zosky and Sly, 2007). Johnson et al. showed that mice continuously exposed to HDM, but not OVA, had demonstrated persistent eosinophilic air-way inflammation which suggests that repeated exposure to OVA could have led to allergic tolerance (Johnson et al., 2004). To test whether the asthma model was successfully set up, we performed BW monitoring, airway hyperresponsiveness measurement, and pulmonary histopathological assessment. In our study, we found that the allergic asthma model mice have some symptoms such as restlessness, dyspnea or irregular breathing rhythm and significant weight loss from the sensitization stage. Pulmonary function test is an important method to diagnose asthma and evaluate the degree of asthma control (Kumar et al., 2008). AHR was found in the HDM treatment group. Infiltration of inflammatory cells in the lungs is also an important characteristic of asthma and can cause an inflammatory and allergic response (McBrien and Menzies-Gow, 2017). Asthma has been classified into various phenotypes, the most well described being eosinophilic asthma (Salter et al., 2019). Numerous inflammatory cells infiltrate around the bronchus (Fahy, 2015). In our study, the pathological manifestations of the lung were bronchial stenosis surrounded by a large number of inflammatory cells (mainly eosinophils).

The significant source of cytokines derived may implicate eosinophils, thereby contributing to airway inflammation (Christianson et al., 2015). So, we next examined cytokines in BALF of mice lung. IL-4, IL-5 and TNF-α play an important role in the infiltration of eosinophils into vascular cell adhesion molecule-1 (VCAM-1) and causes the transendothelial migration of eosinophils (Steinke and Borish, 2001), while IL-5 strongly promotes the maturation adhesion and activation of eosinophils (Matucci et al., 2019). Inhibition of TNF-α can significantly reduce the imbalance of metalloproteinases in asthma (Vieira et al., 2020). We chose to detect IL-4, IL-5, and TNF-α by using Luminex liquid suspension chip. Luminex technology, developed on the basis of ELISA, not only has high specificity through double antibody selection, but also can complete the analysis of a variety of target molecules in a single experiment. In the present study, we found that IL-4, IL-5 and TNF-α concentrations were significantly increased in the HDM group. These results demonstrated the successful construction of the asthma mice model.

To understand the cognitive status of asthmatic mice, we examined the learning and memory abilities of the allergic asthma model mice with Barnes mazes test, which is an important method to evaluate the cognitive learning ability of mice (Pitts, 2018). The Barnes maze offers important advantages worth noting: the Barnes maze does not involve swimming and the potential confounding factors associated with it (Pitts, 2018). So, we think that Barnes maze may be the most suitable choice for learning and memory test in asthmatic mice. Decline in spatial learning and memory was observed in our 60-days allergic asthma model. This result is consistent with previous studies (Guo et al., 2013). This result suggested that the asthmatic mice had a certain degree of cognitive dysfunction.

Cognitive dysfunction refers to deficits in learning and memory, social functioning, language, visuospatial functioning, complex attention, or executive function (Sachdev et al., 2014). Any direct or indirect factors leading to abnormalities in brain function may lead to cognitive dysfunction (Sanford, 2017; Wang et al., 2019). The hippocampus is a brain area critical to learning and memory (Scoville and Milner, 1957). Synapses in the hippocampus damage are among the earliest known pathological changes and the best correlates of memory impairment (Batista et al., 2018). Then we observed the changes of synapses in the brain of all the mice by TEM. In this study, ultra-structural analysis revealed the length of active zone and the number of synaptic vesicles in the asthma group were both significantly smaller than that in the other three groups. These results indicated a breakdown of the synaptic plasticity. Synapses have variability in morphological structure and functional activities, namely synaptic plasticity that include synaptic area, shape, number and gap (Gilbert et al., 2017).

Studies have shown that the synaptic loss and dysfunction due to ischemia and hypoxia could induce spatial learning and memory impairment (Gruneberg et al., 2016). The hippocampus seems more susceptible to hypoxia than the other brain regions (Coimbra-Costa et al., 2017). We also investigated the expression of HIF protein markers in the hippocampus and the cortex, namely, HIF-1α and HIF-2α. HIF is key transcriptional mediators of hypoxia response (Semenza and Wang, 1992). The hypoxia-inducible factor family is mainly composed of HIF-1 and HIF-2, and consists of *α* and *β* subunits. Numerous studies documented that HIF-1α was upregulated in mice treated with hypoxia (Li et al., 2017), but few researchers investigated the expression of HIF-2α in the brain. In the present study, Western blotting and immunofluorescence assay results showed increased expression of HIF-1α in the hippocampus and the cortex of the asthmatic mice. In addition, HIF-2α expression was also up-regulated. HIF-2α are also expressed in human cells under normal oxygen (Jiang et al., 1996). However, the synthesized HIF-2α proteins are quickly degraded by intracellular oxygen dependent ubiquitin-protease degradation pathway, which could only be stably expressed under hypoxia (Loboda et al., 2010). The present results suggested that hypoxia occurred in the brain of asthmatic mice.

Hypoxia is an important factor causing vascular disease, and further vascular changes aggravate hypoxia (Rathnasamy et al., 2015). It has been reported that hypoxia environment can cause many pathological changes of blood vessels (Mansur et al., 2018). Subsequently, we focused on the blood vessels in the brain tissue. We found severe edema and collapse in the blood vessel in the brain of the asthma model mice. Blood vessels can be affected for a variety of reasons, not only by oxygen but also by inflammation (Adrover et al., 2019). The histamine and platelet activating factor secreted in the inflammatory environment could directly shrink the bronchial smooth muscle, and result in spasm and hypoxia (Lemanske and Busse, 2003). So we wondered whether this change was caused by airway inflammation in asthma or by the AHR. We raised the hypothesis that bronchial inflammation caused AHR, which then caused hypoxia.

To test our hypothesis, we used DEX which was delivered as an important glucocorticoid class. There are various methods for the treatment of asthma, among which glucocorticoid therapy is one of the most important methods for the treatment of most asthma (O'Byrne and Mejza, 2018). DEX could reduce the number of eosinophils in people’s peripheral blood and the level of Ig E in serum, thereby reducing the symptoms of asthma patients (Deykin et al., 2005). Airway inflammation is the most characteristic of asthma. Interestingly, our study found that the inflammatory infiltrates in the lungs and the concentrations of inflammatory cytokines in BALF with DEX-treated were reduced. Hypoxia in the lung tissue was improved with the use of DEX. In addition, the brain lesions in the DEX-treated mice were relieved. One limit of the study was that we investigated the effect of DEX on cognitive dysfunction although one of the main objectives of asthma therapy is to avoid oral corticosteroids. In addition, Dexamethasone, by itself, has anti-inflammatory effects in brain cells, can reduce vascular edema, and can have effects on cognitive dysfunction (Karaman et al., 2017). It would be very useful to evaluate the effects of inhaled corticosteroids on the cognitive dysfunction in asthma in order to better demonstrate that the neurological benefits of corticosteroids are mediated by their anti-inflammatory activity on airways.

In summary, we found that asthma caused the impairment of learning and memory, aberrations of synaptic tissues and blood vessels, increased levels of HIF-1α and HIF-2α. This phenomenon suggests that asthma may result in a lack of oxygen and eventually lead to cognitive impairment. These findings provide novel strategies and insights for the intervention of the relationship between asthma and cognitive impairment. Moreover, the research of cognitive impairment in asthmatic patients may provide a therapeutic target to improve the quality of life of patients.

## Data Availability

The original contributions presented in the study are included in the article/Supplementary Material, further inquiries can be directed to the corresponding authors.

## Abbreviations

AHR, airway hyperresponsiveness; BALF, Bronchoalveolar lavage fluid; BCA, Bicinchoninic acid; BW, Body weight; DAPI, Diamidino phenylindole; DEX, Dexamethasone; ECL, Electrochemiluminescence; ELISA, Enzyme-linked immunosorbent assay; GAPDH, Glyceraldehyde-3-phosphate dehydrogenase; HDM, House dust mites; HE, Hematoxylin eosin; HIF, Hypoxia inducible factor; HIF-1α, Hypoxia-inducible factor-1α; HIF-2α, Hypoxia-inducible factor-2α; Ig, Immunoglobulin; IL-4, Interleukin-4; IL-5, Interleukin-5; NS, Normal saline; PBS, Phosphate buffered saline; PMSF, Phenylmethyl sulfonyl fluoride; RAPA, Rapamycin; SDS-PAGE, SDS-Polyacrylamide gel electrophoresis; TEM, Transmission electron microscopy; TNF-α, Tumor necrosis factor-α; VCMA-1, Vascular cell Adhesion molecule-1; WB, Western blotting.
