# Supplementary material for: Allergic Asthma-Induced Cognitive Impairment is Alleviated by Dexamethasone
Source: Front Pharmacol. 2021 Jun 23;12:680815. doi: 10.3389/fphar.2021.680815 (PMC8261293; doi:10.3389/fphar.2021.680815)
Supplement: Supplementary file 1 [file image1.pdf]

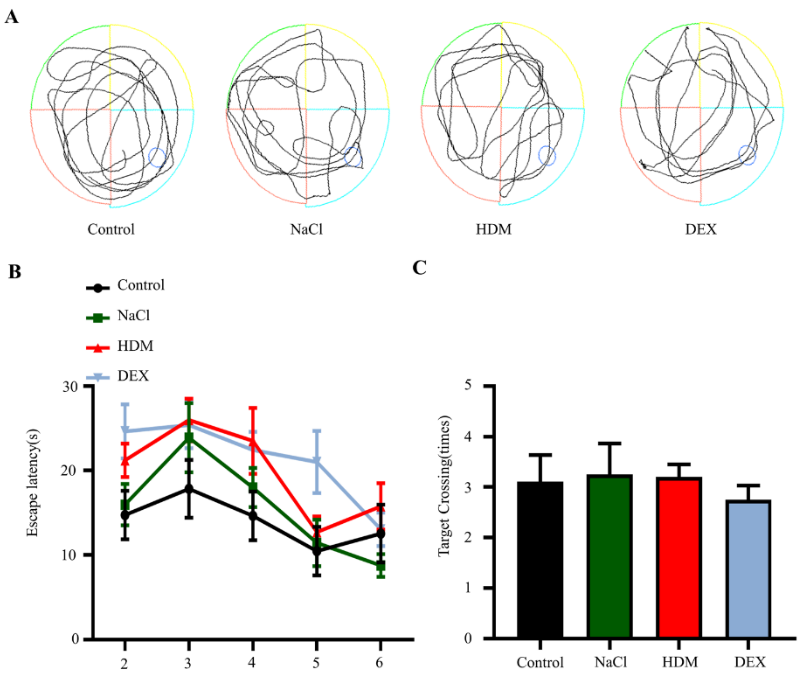

Figure S1

Figure S1 (A) Morris Water maze movement track. **(B)** Lurk time to reach the escape platform on training day in the Morris water maze experiment. **(C)**The number of times the platform area was crossed on the test day in the Morris water maze experiment.
